# Case Report: The nonsense variation of the cardiac transcription factor *NKX2-5* has been identified in a Chinese family with nonsyndromic congenital heart disease

**DOI:** 10.3389/fgene.2025.1498144

**Published:** 2025-07-09

**Authors:** Haixia Zhang, Jing Chen, He Wang, Qinqin Xiang, Shanling Liu

**Affiliations:** ^1^ Department of Medical Genetics/Prenatal Diagnostic Center, West China Second University Hospital, Sichuan University, Chengdu, Sichuan, China; ^2^ Key Laboratory of Birth Defects and Related Diseases of Women and Children, Ministry of Education, Sichuan University, Chengdu, Sichuan, China

**Keywords:** nkx2-5, nonsense variant, trio-whole-exome sequencing, nonsyndromic congenital heart disease, case report

## Abstract

**Background:**

NK2 HOMEOBOX 5(OMIM: 600584, *NKX2-5*), a pivotal cardiac regulatory transcription factor, represents the initial identified genetic etiology underlying congenital heart diseases (CHDs). As a member of the NK homeobox gene family, *NKX2-5* functions as an essential DNA-binding transcriptional activator. It demonstrates robust expression levels in both primary and secondary heart fields’ cardiac progenitor cells, playing an indispensable role in cardiovascular development. Here we reported a *NKX2-5* nonsense variant in a Chinese family with nonsyndromic congenital heart disease.

**Case presentation:**

Trio-whole-exome sequencing (Trio-WES) was performed on the proband and parents, followed by Sanger sequencing for verification and linkage analysis using available DNA samples from this family and additional family members. A nonsense variant (NM_004387.4: c.342C>A, p.(Cys114*)) was identified within the *NKX2-5* gene through Trio-WES analysis and classified as likely pathogenic according to the criteria of the ACMG. Sanger sequencing revealed the presence of this nonsense variant in all affected family members (II1, II3, III1, and III5) within the *NKX2-5* gene, while unaffected family members (II2, II7, and II8) did not exhibit this variant.

**Conclusion:**

The present study identified a heterozygous nonsense variant of the *NKX2-5* gene in a family with nonsyndromic congenital heart disease, suggesting that this variant may be the underlying cause of the disease within this particular family. Our findings suggests that it can cause diverse phenotypes and varying severity of cardiac abnormalities even within the family. Additionally, an early and definitive genetic diagnosis can provide precise information for subsequent treatment and fertility counseling.

## Background

The *NKX2-5* gene is located on chromosome 5q35.1 and consists of two coding exons that encode a protein consisting of 324 amino acids. Similar to other members of the NK2 family of transcription factors, it contains a highly conserved homeodomain (HD), which encompasses a helix-loop-helix domain with three alpha helices responsible for recognizing and binding specific DNA sequences (Chen and Fishman, 1996; Dixit et al., 2021). A transient upregulation of *NKX2-5* expression occurs during conduction system development, indicating a crucial role of this gene in the maturation and establishment of the conduction system through modulation of gap junction and ion channel protein expression (Pashmforoush et al., 2004). Although the association between *NKX2-5* gene variants and various congenital heart diseases is well-established, the genotype-phenotype correlation of these variants in heart diseases remains unclear (Zhu et al., 2000). Our study implemented the genetic test of a large non-syndromic congenital heart disease family in China, with the objective of identifying the underlying cause and providing more precise information for subsequent genetic counseling.

## Case presentation

### Clinical findings

**FIGURE 1:**
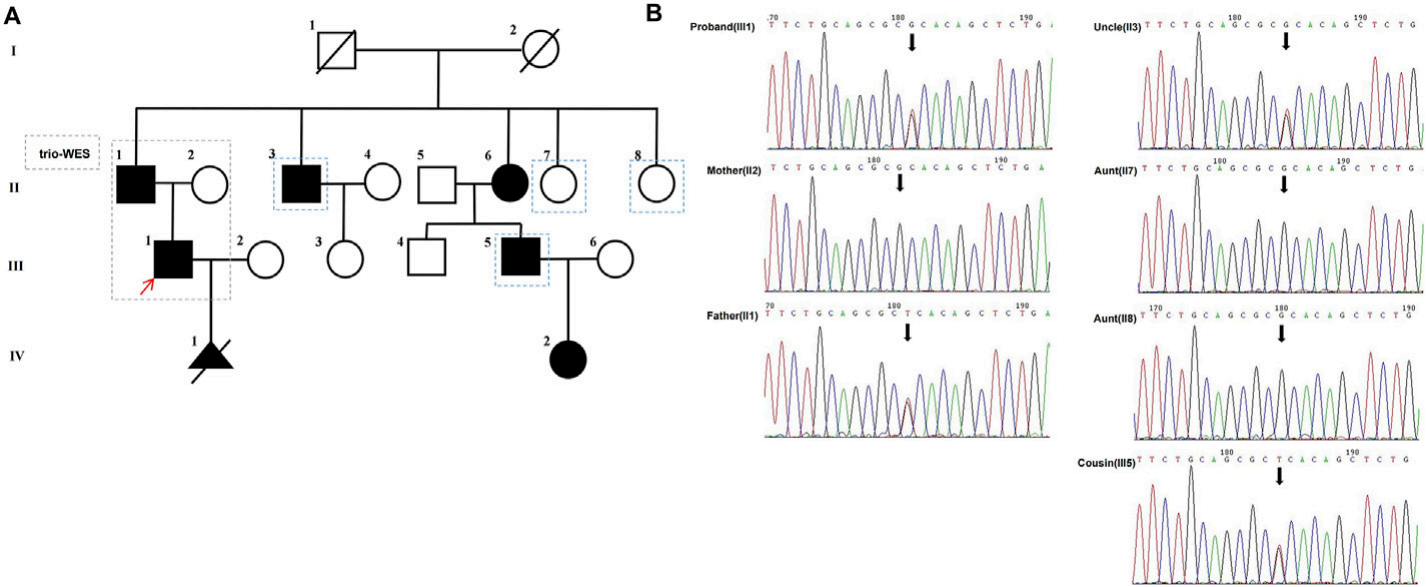
**(A)** The pedigree of the proband’s family is presented with generations labeled as I-IV. Squares represent males, while circles represent females. Filled symbols indicate affected individuals, whereas empty symbols denote unaffected individuals. Deceased individuals are marked with a slash (/), and the red arrow indicates the proband, the dashed blue lines represent family members for verification in Sanger sequencing. **(B)** Verification of the candidate variant (*NKX2-5*, c.342C>A, p.(Cys114*)) of gDNA (II1, II2, II3,II7, II8, III1,III5) by Sanger sequencing.

We collected a Chinese family with segregation of a likely pathogenic variant on NKX2-5 gene that causes nonsyndromic congenital disease (Figure 1).

**FIGURE 2:**
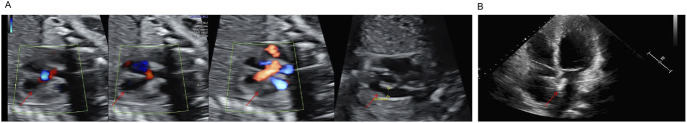
**(A)** Biphasic Shunt at the Fetal Ventricular Level, Overriding aorta, Arterial stenosis. **(B)** The closure of the atrial septal defect.

The proband (III1), a 28-year-old male, primarily presented with an atrial septal defect (ASD) and pulmonary hypertension (PH). At the age of 19, he underwent successful surgical repair for the atrial septal defect (Figure 2B) and achieved satisfactory postoperative recovery. Prenatal ultrasound (Figure 2A) revealed tetralogy of fallot and bilateral ventricular horizontal shunt in the fetus of the proband (III1) and his partner(III2), leading to the termination of the pregnancy (IV1). Regrettably, no sample of the induced fetus(IV1) was available. After undergoing comprehensive genetic counseling, it was discovered that several relatives within the proband’s family exhibited similar phenotypes characterized by cardiac system abnormalities. All participants received diagnoses through echocardiography. The family pedigree is shown in Figure 1A, while Table 1 presents comprehensive clinical characteristics of patients within this family.

**TABLE 1:** The clinical features observed in individuals diagnosed with congenital heart disease in this family.

Pedigree	Atrial septal defect	Other structural cardiac defects	Comment
II1	-	Patent foramen ovale, Pulmonary artery stenosis, Biatrial enlargement, Dilated right ventricle, Interatral septal aneurysm, Mitral regurgitation, Tricuspid Regurgitation, Pulmonary regurgitation	
II3	+	Mitral regurgitation, Tricuspid Regurgitation, Pulmonary regurgitation, Aortic regurgitation, ventricular wall motion abnormality	
II6	+	N/A	
III1	+	Pulmonary hypertension	Surgical repair for the atrial septal defect
III5	+	N/A	
IV1	-	Tetralogy of Fallot	Fetus-induced
IV2	+	N/A	

Written informed consent was obtained from this family with the approval of the Ethics Committee at West China Second Hospital of Sichuan University (2024–228).

### The analysis of whole exome sequencing and sanger sequencing

Trio-WES of II1, II2 and III1 was performed as previously described using an Illunima NovaSeq6000 platform (Chen et al., 2022). Sequencing reads were aligned to the reference human genome GRCh38/hg38 using Burrows-Wheeler Aligner (BWA, v0.7.17). The ENLIVEN variants annotation interpretation system (Berry genomics) were used for functional annotation. The databases utilized for annotations mainly included gnomAD_exome(v2.1.1), gnomAD_genome(v3.1), ExAC(v1), 1000 Genomes Project (1000 G) (phase3), Human Gene Mutation Database(HGMD^®^, 2024.3), Online Mendelian Inheritance in Man(OMIM^®^, 20241110),ClinVar (20241111), Combined Annotation Dependent Depletion(CADD, v1.7), dbNSFP(v4.7a). The candidate likely pathogenic variants associated with nonsyndromic congenital heart disease were assessed according to the guidelines provided by the ACMG (Richards et al., 2015).

The variant revealed through Trio-WES was confirmed by Sanger sequencing, and family co-segregation analysis was conducted for members of this family who willing to provide DNA samples. The forward and reverse primers utilized for Sanger sequencing analysis are presented below: *NKX2-5*-Forward: 5′-ATC​TTG​ACC​TGC​GTG​GAC-3′ and *NKX2-5*-Reverse: 5′-CTT​GAG​CCA​GCC​TGA​CTT-3’. The PCR products were subsequently subjected to sequencing analysis using an ABI 3500 Genetic Analyzer (Thermo Fisher Scientific) in order to validate the presence of the variant at c.342C>A in the *NKX2-5.*

### Genetic findings

A nonsense variant (NM_004387.4: c.342C>A, p.(Cys114*)) was identified within the *NKX2-5* gene through Trio-WES analysis conducted on the proband (III1) and his parents (II1, II2).

Sanger sequencing was performed on all available DNA samples from family members (II1, II2, II3, II7, II8, III1 and III5) for verification and linkage analysis. The results showed that the NKX2-5 nonsense variant was present in all affected family members (II1, II3, III1 and III5), but not in unaffected family members (II2, II7 and II8) (Figure 1B).

**FIGURE 3:**
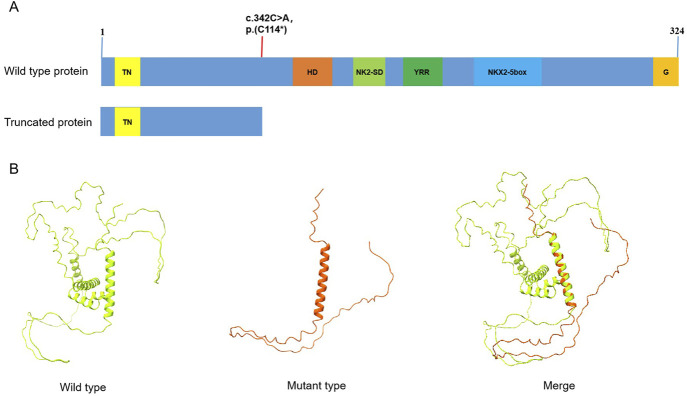
**(A)** The domain distribution of NKX2-5 gene, and the red line shows the location of variation in the article, the variant (c.342C>A) in the NKX2-5 gene leads to premature transcription termination, resulting in a truncated protein at position Cys114*; TN: tinman domain; HD:homeodomain; NK2-SD:NK2-specific domain; YRR:tyrosine-rich region; G:GIRAW. **(B)** The 3D model (AlphaFold2) based on NKX2-5 protein sequence, Comparison of wild-type and truncated protein. Green model: wild type; red model: a heterozygous variant in the NKX2-5 gene (NM_004387.4: c.342C>A, p.(C114*).

The variant (c.342C>A) was not found in control databases such as the 1,000 Genomes Project database, ExAC, and gnomAD. The ClinGen haploinsufficiency (HI) score of *NKX2-5* is 3, suggesting that there was sufficient evidence of haploinsufficiency in this gene. The transcript NM_004387.4 has two exons, and the variant(c.342C>A) is located on the last exon. Generally, Nonsense-Mediated Decay (NMD) is not predicted to occur if the premature termination codon occurs in the 3′ most exon or within the 3′ most 50 nucleotides of the penultimate exon, so the nonsense variant p.(Cys114*) is predicted to truncate the protein after 114 amino-acid (Figure 3A) and may loss of all crucial functional domains associated with cardiac transcription factors. The 3D model (Alphafold 2) based on NKX2-5 protein sequence indicated this nonsense variant may lead to the deletion of most of the protein sequence of the gene (Figure 3B). It is highly likely that this variant leads to a complete loss of NKX2-5 function, indicating haploinsufficiency as the underlying pathophysiology in this nonsyndromic congenital heart disease family. Furthermore, based on the ACMG criteria, this variant (c.342C>A) has been classified as “Likely Pathogenic” (PVS1_Strong(This nonsense variant is not predicted to undergo NMD but truncated/altered region is critical to protein function)+PM2_supporting(This variant is absent from controls)+PP1_Moderate(This variant is cosegregation with disease in multiple affected family members), (The total score is 7 points) (Tavtigian et al., 2020).

## Discussion and conclusion

The heart is the first organ to form during embryonic development and is an essential prerequisite for embryo growth and survival, as it provides adequate oxygen and nutrients through the circulatory system. The formation of a fully functional four-chamber heart results from the precise coordination of cell differentiation processes that integrate multicellular morphogenesis in the early embryo. CHDs are the most common birth defects in humans, with an estimated prevalence of up to 1% for live births and 10% for stillbirths (Xu et al., 2017; Akazawa and Komuro, 2005; Fahed et al., 2013; Marelli et al., 2014; Mozaffaria et al., 2015).

Cardiac development is a complex biological process that necessitates the integration of cellular commitment, morphogenesis, and excitation-contraction coupling. Numerous empirical findings have demonstrated the indispensable role of *NKX2-5* in regulating septal formation and facilitating the maturation and maintenance of atrioventricular node function during cardiac morphogenesis (Pashmforoush et al., 2004; Bruneau, 2008; Harvey, 2002). Consequently, genetic variations in *NKX2-5* can lead to nonsyndromic congenital heart disease (Schott et al., 1998). The association between genetic variants of the *NEX2-5* gene and cardiovascular abnormalities has been confirmed in various animal models, including *Drosophila*, frog, and mouse (Tanaka et al., 2002; Harvey, 2002). Knockout of the *NKX2-5* gene can cause cardiovascular abnormalities and impaired heart development.

Variants in *NKX2-5* gene can cause various congenital heart defects, indicating that this transcription factor is involved in multiple pathways of heart development, including atrial, ventricular, and septal development, as well as atrioventricular conduction and atrioventricular valve formation (Benson et al., 1999). The families in the present study exhibited diverse cardiac abnormalities, similar to those observed previously.

The expression of *NKX2-5* occurs in the heart and cardiac progenitor cells during early stages of development when the two cardiac primordia are symmetrically positioned in the anterolateral mesoderm. Although different nonsense variants of the *NKX2-5* gene lead to the same cardiac abnormal phenotype, there are differences in transcriptional activation ability and DNA binding ability among various variants. The apoptosis of myocardial cells may be one of the reasons why certain nonsense variants induce heart defects (Zhu et al., 2000).

The *NKX2-5* gene variants have been identified in numerous cardiac disorders, including tetralogy of fallot, nonsyndromic CHDs, ASD, patent foramen ovale, and others. Although the association between *NKX2-5* gene variants and various congenital heart diseases is well-established, the genotype-phenotype correlation of these variants in heart diseases remains unclear (Goldmuntz et al., 2001; Gioli-Pereira et al., 2010; Belvís et al., 2009; Watanabe et al., 2002). The Chinese family predominantly exhibit atrial septal defect in our study, while other cardiac abnormalities observed include tetralogy of fallot, and patent foramen ovale, etc. The same nonsense variant (NM_004387.4: c.342C>A, p.(Cys114*)) in N*KX2-5* gene is associated with variable disease severity and cardiac phenotype even within the family. Growing evidence supports oligogenic and multifactorial models of CHDs (Gifford et al., 2019). However, since our study only performed Trio-WES on the proband and parents, there are limitations in analyzing other genetic factors beyond the rare likely pathogenic variant in this family.

This variant has been submitted to the ClinVar database and is associated with atrial septal defect 7 (ASD7), where it has been classified as pathogenic (Accession:RCV002889896.2). However, this variant has not been reported in the literature in individuals affected by NKX2-5-related disorders. In addition, the ClinVar database also includes other variants in the NKX2-5 gene that affect the same amino acid(NM_004387.4:c.342C>G(p.Cys114Trp); c.340T>G(p.Cys114Gly)), but these are all classified as uncertain significance (VUS) and lack supporting literature reports. Additionally, the gnomAD database contains LoF variants for NKX2-5 gene. This observation may be attributed to variable expressivity of the gene-related phenotypes, suggesting that some asymptomatic individuals might have been included in the control population database. Notably, the two variants rs1761374435 and rs1761372394, annotated as loss-of-function (LoF) in gnomAD, show relatively high population carrier frequencies (0.0003431 and 0.0001467 in East Asian populations, respectively). Through UCSC genome browser analysis, we identified that both variants are located within intronic regions of the MANE-select transcript, suggesting their functional impact may be limited.

To date, the HGMD Professional database has documented loss-of-function variants in the NKX2-5 gene across dozens of cases involving various cardiovascular disorders, suggesting sufficient evidence of haploinsufficiency for this gene. The NKX2-5 protein contains an evolutionarily conserved HD domain located at amino acid positions 145 to 197, which specifically functions to recognize and bind consensus DNA motifs. In this present study, a heterozygous variant of *NKX2-5*(NM_004387.4: c.342C>A, p.(Cys114*)), was identified in a family with nonsyndromic congenital heart disease, which is expected to result in the production of a truncated protein. Therefore, it is plausible to consider this variant as the underlying cause of the disease within this particular family. However, the specific impact of this variant on the protein requires further functional experimental validation. For example, the expression of truncated transcripts can be confirmed through transcript data (such as RT-PCR), and the loss of transactivation activity can be assessed via functional experiments (such as luciferase reporter assays).

Small ASD may close spontaneously during infancy or childhood, while large ASD in adulthood may lead to massive blood shunts from left to right, leading to congestive heart failure, atrial arrhythmia, sudden cardiac death and other serious abnormalities. The ultimate therapeutic approach for certain patients involves heart transplantation, and a precise genetic diagnosis holds great significance in terms of treatment and subsequent guidance on fertility (Ellesøe et al., 2016; van der Meulen et al., 2022).

The findings suggest that the same variant may manifest diverse phenotypes and varying severity of cardiac abnormalities even within the family. An early and definitive genetic diagnosis can provide precise information for subsequent treatment and fertility counseling.

## Data Availability

The variation data reported in this paper have been deposited in the Genome VariationMap (GVM) (https://ngdc.cncb.ac.cn/gvm/) in National Genomies Data Center, Beijing Institute of Genomics, Chinese Academy of Sciences and China National Center for Bioinformation, under accession number GVM000826.

## Abbreviations

NKX2-5: NK2 HOMEOBOX 5, CHDs: congenital heart diseases, Trio-WES: Trio-whole-exome sequencing, ACMG: American Society for Medical Genetics and Genomics, HD: homeodomain, ASD:Atrial septal defect, PH: Pulmonary hypertension.
